# Methodological study of affine transformations of gene expression data with proposed robust non-parametric multi-dimensional normalization method

**DOI:** 10.1186/1471-2105-7-100

**Published:** 2006-03-01

**Authors:** Henrik Bengtsson, Ola Hössjer

**Affiliations:** 1 Mathematical Statistics, Centre for Mathematical Sciences, Lund University, Box 118, SE-221 00 Lund, Sweden; 2 Mathematical Statistics at the Mathematics Department, Stockholm University, SE-106 91 Stockholm, Sweden

## Abstract

**Background:**

Low-level processing and normalization of microarray data are most important steps in microarray analysis, which have profound impact on downstream analysis. Multiple methods have been suggested to date, but it is not clear which is the best. It is therefore important to further study the different normalization methods in detail and the nature of microarray data in general.

**Results:**

A methodological study of affine models for gene expression data is carried out. Focus is on two-channel comparative studies, but the findings generalize also to single- and multi-channel data. The discussion applies to spotted as well as in-situ synthesized microarray data. Existing normalization methods such as curve-fit ("lowess") normalization, parallel and perpendicular translation normalization, and quantile normalization, but also dye-swap normalization are revisited in the light of the affine model and their strengths and weaknesses are investigated in this context. As a direct result from this study, we propose a robust non-parametric multi-dimensional affine normalization method, which can be applied to any number of microarrays with any number of channels either individually or all at once. A high-quality cDNA microarray data set with spike-in controls is used to demonstrate the power of the affine model and the proposed normalization method.

**Conclusion:**

We find that an affine model can explain non-linear intensity-dependent systematic effects in observed log-ratios. Affine normalization removes such artifacts for non-differentially expressed genes and assures that symmetry between negative and positive log-ratios is obtained, which is fundamental when identifying differentially expressed genes. In addition, affine normalization makes the empirical distributions in different channels more equal, which is the purpose of quantile normalization, and may also explain why dye-swap normalization works or fails. All methods are made available in the aroma package, which is a platform-independent package for R.

## Background

The objective of most gene-expression measurements is to assess the *expression levels *of (all or a subset of) genes in one or several cell populations. Typically, mRNA abundances are measured, although techniques for measuring protein-levels also exist. The *microarray technique *[1] provides a way to measure mRNA transcripts for a large number of genes simultaneously, typically in the order of 10^3 ^– 10^5 ^or more. Microarrays have well defined immobilized regions, which each consists of clones or synthesized sequences of DNA specific to a unique gene. We refer to these (non-hybridized) regions or spots as *probes *[2]. A cocktail of cDNA created from the RNA extract from the cell population in study is then, for a few hours, *hybridized *to the DNA on the microarray after which excess cDNA is washed off. The result is that each region of the microarray contains a certain amount of hybridized DNA unique to the corresponding gene. By first labeling the cDNA strands in the sample cocktail with a radioactive or a fluorescent probe, the amount of hybridized DNA can be measured utilizing radioactive sensitive film or a color-sensitive scanner, respectively.

By measuring the gene expression for a specific gene, we try to assess how active that gene is (measured on some scale). Because it is hard to identify an *absolute *scale to measure on, often, but also for various other reasons, a reference is used to obtain a *relative *scale. As even genes from the same sample are not directly comparable to each other, each gene gets its own reference, which is typically the same gene from a reference sample. With this approach, we can obtain *gene-expression ratios *for every gene, which for instance can be used to test the hypothesis if a gene (in the test sample) is *differentially expressed *or not (compared to the gene in the reference sample). This is the core idea behind the two-channel microarray technology, in which the test and the reference cDNA cocktails are hybridized simultaneously and in a competitive way to the same array. The same idea has been adopted by single-channel hybridization technologies where the comparison instead is done numerically in the data analysis step. Even if gene-by-gene references are used, the measurements are not perfect and they are likely to contain systematic errors, which possibly vary from measurement to measurement, and the obtained gene-expression ratios may still be biased and not comparable to each other. What we ultimately would like to do is to measure all control and all reference samples under identical conditions. The aforementioned two-color microarray technology tries, in some sense, to do this by measuring the control/reference pairs for each gene in one hybridization (although it is not clear if the gain from co-hybridizing two samples with different labels is larger than hybridizing twice with identical labels and then scanning the samples separately).

In this paper, we present an affine model that explains many of the systematic effects frequently observed when gene-expression levels from two (or more) samples are compared. The main contributors to such systematic effects are offsets in the individual channel signals, which give non-linear systematic effects in ratios. We will not provide an error model, but only a deterministic model. The main reason for this is that an error-free model makes it easier to understand the impact that channel offsets have on the downstream analysis regardless of gene-expression technology used. This is especially of interest as these are often implicitly assumed to be small and of no effect, which we believe is a too strong assumption. The impact of channel offsets is much larger that the noise, which is why we allow us to assume zero noise in the discussion. Although some error models have been suggested for microarray data [3], we believe research beyond this article is required before we can understand and correctly model the various error sources introduced in the microarray process.

The outline of this paper is as follows. In the Model section, a general model that incorporates all steps of any gene-expression technology is given. By dissecting the generic model and focusing more on the microarray technologies, an affine model is introduced. Here is also the widely adopted and accepted log-ratio log-intensity transform under affine transformations formalized. The Results section consists of three main parts. In the first, we show how the affine transform introduces intensity and fold-change dependent biases in the log-ratios. In the second part, we revisit common normalization methods, to which dye-swap and background correction may also be counted, and discuss them using the affine model. In the third and concluding part, we suggest a novel and multi-purpose robust normalization method to back-transform data to the linear (proportional) space. We end the paper with a Discussion section where we give similarities to other normalization methods followed by a Conclusions section. Details on calculations and the data set used are given in the Methods appendix.

## Results

### General model

Consider an experiment with genes *i *= 1,..., *I *from RNA extracts *c *= 1,..., *C*. For example, in oligonucleotide microarrays each slide measures the gene-expression levels of exactly one RNA extract whereas for a two-color microarrays each slide measures two RNA extracts, one in each channel. From now on, we refer to the RNA extracts or replicates of such as *channels*. Let *x*_*c,i *_be the true gene-expression level of gene *i *in channel *c *and let *y*_*c, i *_be the corresponding observed gene-expression level. The relationship between the observed and the true expression levels can be written as

*y*_*c,i *_= *f*_*c*_(*x*_*c,i*_) + *ε*_*c,i *_    (1)

where *f*_*c *_is a channel specific *measurement function*, which includes all steps in the gene-expression acquisition process. Most generally, we have that *E *[*ε*_*c,i*_] = 0 and *V *[*ε*_*c,i*_] = $\sigma_{c,i}^{2}$, where the variance can take any form. Importantly, the properties of *ε*_*c *_are not well understood and depends on platform used, but also which part of the process that is studied. For this reason and because of the many interesting effects that the affine transformation (presented below) generates by itself, we conduct this study under the assumption of noise-free data. Relationship (1) may be specified for subsets of genes or probes, e.g. print tip [4], microtiter plate or clone library [5] groups. Spatial dependencies may also be modeled. However, to simplify the discussion that follows, we avoid such details.

Since inference is ideally based on *x*_*c*,*i*_, the inverse of *f*_*c *_has to be identified, something that, in theory, is possible if it is strictly increasing. Violation of this constraint has been observed in, for instance, two-color microarray data. This can be due to too high concentrations of fluorophores, which sometimes quenches the signal so much that the signal decreases when the concentration increases [6,7]. Extreme saturation in the scanner, which is commonly observed when the PMT gain is set too high, results in censored signals, which in turn prevents a unique inverse of the measurement function to be found. This paper does not discuss saturation further, because we believe that saturation can and should be avoided.

### Dissection of the overall measurement function

Formally, each step in the microarray process can be seen as a function that takes a set of input objects and outputs another set of objects. The sequential nature of the process makes it possible to think of the measurement function *f*_*c *_as a *composite function *(function of functions); *f*_*c *_= *f*_*c*,*S *_◦ *f*_*c,S*-1 _◦ ⋯ ◦ *f*_*c*,1_, where *S *is the number of steps in the process. For instance, and of course simplified, it could be that *f*_*c*,1 _models the extraction of the RNA from the cell, *f*_*c*,2 _models the reverse transcription of RNA into cDNA and so on. Some of these *submeasurement functions *are shared by several channels and others are channel specific or even gene specific. Moreover, there may be joining subfunctions too, e.g. the hybridization of labeled cDNA sequences to the probes on the array. In this paper, measurement functions of different channels are treated independently.

A first-order Taylor series expansion of an arbitrary measurement function *f*_*c*_(*x*_*c,i*_), has the form

*f*_*c*_(*x*_*c,i*_) = *a*_*c *_+ *b*_*c*_*x*_*c,i *_+ *R*_*c*_(*x*_*c,i*_), ∀*c,i*.     (2)

From the above dissection of a measurement functions, it is easy to argue that some of the subfunctions may introduce offset (bias) and that there for this reason ought to be an offset in *f*_*c *_(we will use the terms bias and offset interchangeably). For instance, the offset terms may be due to non-uniformity of the reverse transcription, the labeling [7] or the hybridization, due to dark noise in the PMT [8] or laser scatter light in the scanner, background noise, non-uniformity of the scanned glass slide [9], or threshold effects etc. In [10] it is shown how various background estimates based on different image analysis methods may introduce bias. Similarly, we have shown that different scanners may introduce bias [11].

### The affine measurement function

In order to focus on the effects of *a*_*c *_and *b*_*c*_, but also because it results in the simplest parametric measurement function possible, we assume *R*_*c*_(*x*_*c,i*_) in (2) to be small. The *affine measurement function *is

*f*_*c*_(*x*_*c,i*_) = *a*_*c *_+ *b*_*c*_*x*_*c,i*_, ∀*c,i*,     (3)

with unique inverse

$x_{c,i}=f_{c}^{-1}(y_{c,i})=\frac{y_{c,i}-a_{c}}{b_{c}},\;\forall c,i,\;\left(4\right)$

where *a*_*c *_is the overall offset (bias) and *b*_*c*_*> *0 is the overall scale factor in channel *c*. The *a*_*c *_parameters are commonly positive, but under certain circumstances, for instance, as demonstrated later, when two different measuring techniques are compared, the effective offset may be negative. Modeling microarray data by an affine transform is not novel [3,12-14], but the reasons for it might have been different in those papers.

### The log-ratio log-intensity transform

In two-color but also in oligonucleotide microarray experiments, it is convenient to do statistical analysis on the log-ratios and the log-intensities [15] of the gene-expression levels in two channels instead of on the expression levels directly. For gene *i *we have that

$M_{i}=\log_{2}\frac{y_{R,i}}{y_{G,i}}=\log_{2}\frac{f_{R}(x_{R,i})}{f_{G}(x_{G,i})}\;\left(5\right)$

$\begin{array}{c} A_{i}=\frac{1}{2}\log_{2}(y_{R,i}\cdot y_{G,i})\\ =\frac{1}{2}\log_{2}(f_{R}(x_{R,i})\cdot f_{G}(x_{G,i})). \end{array}\;\left(6\right)$

For simplicity, we denoted channels 1 and 2 by *R *and *G*, which are mnemonics for the red and the green dyes commonly used in two-color microarray data. A rationale for this bijective transform (if the observed signals are positive) is that the main measure of interest, the fold change, is contained in one variable. However, since the transform is based on observed expression levels and not the true ones, *M *alone does indeed not carry all information about the biological fold change. This can be seen if the *true fold change *for an arbitrary gene *i *is considered;

*r*_*i *_= *x*_*R,i*_/*x*_*G,i *_    (7)

where *r*_*i *_> 0. Dropping gene index *i *in (5) and (6), *M *and *A *can be written as functions of *x*_*G *_and *r*, i.e. *M *= *g*_*r*_(*x*_*G*_) and *A *= *h*_*r*_(*x*_*G*_). Thus,

$M=m_{r}(A)=g_{r}(h_{r}^{-1}(A)),\;\left(8\right)$

which shows that *M *is a function of *A *(and *r*). Hence, and discussed thoroughly below, commonly observed intensity-dependent effects in the log-ratios may contain valuable information, and consequently, applying normalization methods without care may result in loss of information and introduced bias.

### Log-ratios as a function of log-intensities with affine transformations

Under an affine transformation, the relationship between the observed log-ratios and the observed log-intensities for a fixed fold change *r*, omitting gene index *i*, is

$\begin{array}{c} M=m_{r}(A)=\log_{2}r+\log_{2}\beta \\ +\log_{2}\frac{\frac{1}{2}\alpha (r)+\sqrt{\frac{1}{4}{\left[\alpha (r)\right]}^{2}+r\beta 2^{2A}}}{-\frac{1}{2}\alpha (r)+\sqrt{\frac{1}{4}{\left[\alpha (r)\right]}^{2}+r\beta 2^{2A}}} \end{array}\;\left(9\right)$

where *α*(*r*) = *a*_*R *_- *rβa*_*G *_quantifies how much *M *depends on *A *at the given fold change, and *β *= *b*_*R*_/*b*_*G *_is the *relative scale factor *between the two channels compared. See Methods for details. Recall that log_2_*r *is the variable of interest. The derivative of *M *with respect to *A *for a fixed fold change *r *is

${\frac{dM}{dA}|}_{x_{R}=rx_{G}}(A)=-\frac{\alpha (r)}{\sqrt{\frac{1}{4}{\left[\alpha (r)\right]}^{2}+r\beta 2^{2A}}}.\;\left(10\right)$

**Figure 1:**
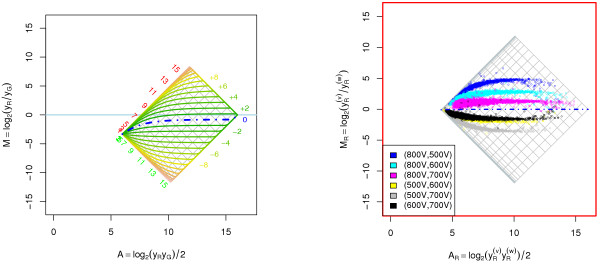
**Affine transformation of the red and the green signals**. *Left*: Affine transformation of the red and the green signals for $A_{1}$ = {(*a*_*G*_, *a*_*R*_) = (200, 20), (*b*_*G*_, *b*_*R*_) = (1.4, 0.8)}. The observed log-ratios as a function of the observed log-intensities for different fold changes. The blue dot-dash curve corresponds to the non-differentially expressed genes and the thinner curves above and below this curve represent log_2_*r *= ± 1, ± 2,... as labeled to the right of the curves. The lines in the gray grid, which is rotated 45 degrees (in (2*A, M*)), show the levels where the *true *signals log_2 _*x*_*R *_and log_2 _*x*_*G *_are equal to ..., -1, 0, 1,..., 16. These levels have been labeled to the left of the grid. No observations can lie outside this grid. *Right*: Real-world example of an affine transformation. The same slide was scanned four times at four different PMT settings. For each of the six scan pairs, the *within-channel *log-ratio and log-intensities were calculated. Data shown is from the red channel, which was estimated to have an offset of *a*_*R *_= 20.3 for all scans.

Consider a fixed *r *and define *α *= *α*(*r*). Then there are only two parameters in (9) and (10) that determine the shape of *m*_*r *_(*A*), namely *α *and *β*. Consequently, when *a*_*R*_, *a*_*G *_≠ 0, *M *is independent of *A *if and only if *α *= 0, that is, when *r *= (*b*_*G*_*a*_*R*_)/(*b*_*R*_*a*_*G*_). For this particular value of *r*, we have that the observed log-ratio is *M *= log_2 _(*a*_*R*_/*a*_*G*_), which is independent of scale factors. Moreover, for log-ratios of non-differential expressions, that is *log*_2_*r *= 0, to be independent of *A*, it must be true that *b*_*G*_*a*_*R *_= *b*_*R*_*a*_*G *_or, equivalently, *b*_*R*_/*b*_*G *_= *a*_*R*_/*a*_*G*_. It is also clear from (10) that the scale parameters cannot introduce any curvature themselves, but only enhance or decrease curvature introduced by the offset. In addition to this, relative scale different from one shifts the log-ratios up or down. Moreover, the size of the effect that the offset terms have on the log-ratios decreases as the intensity increases. At high intensities the only observable effect is that from the relative scale between the two channels. The observed log-ratio for non-differentially expressed genes at high intensity is *M*_∞ _≈ log_2_*β*. In the case of a linear transform (*a*_*R *_= *a*_*G *_= 0), *α *is (always) zero and *M *is therefore independent of *A *for *all r*. The remaining log-ratio bias is log_2_*β*. If *a*_*R*_, *a*_*G *_> 0, the "weakest" observable data point is (*A*_0_, *M*_0_) = (1/2·log_2_(*a*_*R*_*a*_*G*_), log_2_(*a*_*R*_/*a*_*G*_)), which is independent of both gene expression and scale parameters. All fold-change curves converge to this point. In the left graph of Figure 1 the effect of the affine transform $A_{1}$ = {(*a*_*G*_*a*_*R*_) = (200,20), (*b*_*G*_, *b*_*R*_) = (1-4, 0.8)} at different fold changes is depicted. The different curves plotted are the functions *M *= *m*_*r*_(*A*) for different fold changes. Note the asymmetry in curvature between up and down regulation. From the above discussion we know that the observed log-ratios are independent of the log-intensities for *log*_2_*r *≈ -2.51 with value *M*_0 _≈ -3.32. The log-ratio for non-differentially expressed genes at high intensities is *M*_∞ _≈ -0.81. A real-world example taken from [11], where the same array was scanned four times at various scanner PMT (sensitivity) settings, is shown in the right plot of Figure 1. Observed within-channel log-ratios *M*_*c *_= log_2_($y_{c}^{\left(v\right)}$/$y_{c}^{\left(w\right)}$) are plotted against the within-channel log-intensities *A*_*c *_= log_2_($y_{c}^{\left(v\right)}$$y_{c}^{\left(w\right)}$) /2 for the red channel (*c *= *R*) where $y_{c}^{\left(v\right)}$ and $y_{c}^{\left(w\right)}$ are observations at two different scanner PMT settings. In this case it turned out that all scans share the same offset. For more details, see [11]. For another example, see Figure 9.

#### Bias in the log-ratios

**Figure 2:**
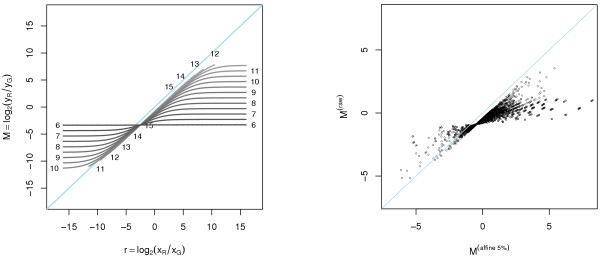
**Bias in the log-ratios introduced by the affine transform**. *Left*: Bias in the log-ratios introduced by the affine transform $A_{1}$. Each line displays the relationship between the observed and the true log-ratios at a certain (observed) log-intensity *A*. Each curve is marked with the value of *A*. We have chosen to truncate the curves when the signals become saturated and the labels for those curves are positioned approximately where they have been truncated. For low intensities there is a great bias (deviance from the diagonal line), especially for large fold changes. At higher intensities the bias is smaller. The curves intersect at the one fold-change level that is independent of the intensity. *Right*: Real-world example of log-ratios for non-normalized versus affine normalized (with 5% negative) signals. The affine parameters are (${\hat{a}}_{G}$, ${\hat{a}}_{R}$, log_2_$\hat{\beta }$) = (45.7, 27.0, -0.418). To clarify the intensity-dependent effect only data points close to *A *= 0.0, 0.5,...,16 are shown.

From (9) we see that the bias in the log-ratios introduced by the affine transform is intensity dependent. This non-linearity can be observed as a propeller shaped graph in Figure 2, where the log-ratios under the affine transform $A_{1}$ are plotted against the true log-ratios at different log-intensity levels. If a regression line is fitted between the affine transformed log-ratios and the true log-ratios, the slope will always be *less *than one. Moreover, this is true for all normalization methods that do not overcompensate for channel offsets. This may explain why some studies show that cDNA microarrays tend to compress the absolute log-ratios compared to oligoarrays and QRT-PCR [16-18] including a recent study [19]; the channel offsets in cDNA microarrays are probably larger. When [20] compared cDNA microarray log-ratios to Northern blot log-ratios for their background correction method they found similar behavior, which emphasizes the close relationship between offset and background estimates. We will return to this later. The same patterns is seen in an *M *versus *M *scatter plot for non-normalized versus (affine) normalized data. See right scatter plot in Figure 2. To visualize the intensity dependency of the log-ratios, only data points at certain log-intensity levels are plotted. For details on data, see Methods.

### Normalization in general

Depending on the design of the microarray experiment, we expect to observe different types of patterns in data. A typical example is where a subset of the genes studied is expected to be non-differentially expressed in a test sample compared to a reference sample. However, it is common that the patterns of the *observed *expression levels are not in line with the expected patterns of the *true *expression levels. Whenever this happens various strategies can be adopted in order to make the normalized data meet the expectations. Normalization of microarray data is about identifying and removing such artifactual variations that are not due to noise or natural variability. An example is the intensity-dependent log-ratio artifact.

In the following section we will, with the affine model in mind, revisit various more or less well known normalization methods that directly or indirectly remove intensity-dependent artifacts. With the gained knowledge, we then propose a generic and robust multi-dimensional normalization method for affine transformed data.

To be more precise in what follows, we will refer to methods that correct for differences in observed and expected data, that is, conform the signals to a standard or a norm, as *normalization methods*, where normalization has the meaning of conforming to expectations. Sometimes *calibration data*, also known as control data, which contains true relative or absolute expression levels, is available. Such data can be used to correct for discrepancies between observed and true expression levels. We refer to methods that use calibration (read *known*) data points to correct for artifacts as *calibration methods*. To this category we also count methods that are based on models for which we can find the inverse of the measurement function. For precise definitions, see the introduction of [21]. Calibration methods are not discussed further in this paper.

Typically a normalization method is only capable of estimating *α *= *a*_*R *_- *βa*_*G *_for *r *= 1 in (9) and not the individual offset terms. This is because the often used *assumption that most genes are non-differentially expressed *(and/or that there is an equal amount of up and down regulated genes) will only help us identify one fold-change curve, namely log_2 _*r *= 0. For a normalization method, like most calibration methods, to be able to estimate both *a*_*R *_and *a*_*G *_more constraints are needed and without known data this can only be done based on more assumptions. As more research is needed, we will not elaborate on such additional assumptions in this paper. Thus, the rest of this paper will only discuss normalization methods based on the commonly accepted assumption that it is possible to identify a set of genes that can be used to normalize the non-differentially expressed genes.

### Curve-fit normalization revisited

When [4] first observed the intensity-dependent effects on the log-ratios they suggested a curve-fit normalization method that is often referred to as *lo(w)ess normalization*. The simplest version of this assumes that the majority of the genes are non-differentially expressed regardless of expression level and for this reason the log-ratios are expected to be centered around zero for all intensities. Under the above assumption, curves estimated using robust local regression methods such as lowess [22,23] or loess [24], or curves modeled by smoothing splines [25] will be good approximations for the *m*_*r *= 1_(*A*) function, which then can be subtracted from the observed log-ratios

*M *← *M *- *m*_*r*=1 _(*A*) = *m*_*r *_(*A*) - *m*_*r*=1_(*A*).     (11)

**Figure 3:**
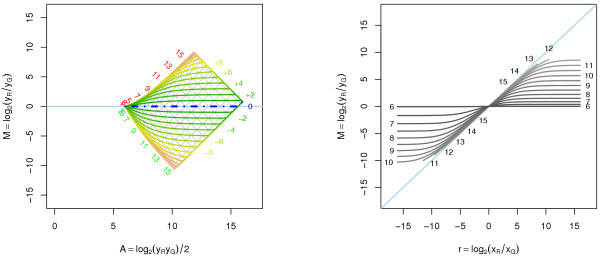
**Curve-fit normalization of affine transformed data**. Curve-fit normalization of $A_{1}$ transformed data. *Left*: Log-ratios as a function of log-intensities for different fold changes. Note that the distance between up- and down-regulated genes at any intensity is the same before and after the normalization. *Right*: Normalized log-ratios versus true log-ratios. We see that intensity-dependent artifacts have been removed for the observed and true log-ratios where all curves intersect (here at (0, 0)).

Under an affine transform, *m*_*r*_(*A*) and *m*_*r*=1 _(*A*) are as in (9), but we do not know of a closed form expression for (11). An example of a curve-fit normalization under the affine transform is depicted in Figure 3. Note that the asymmetry between up- and down-regulated genes is *not *corrected for. Moreover, if we look at the overlaid (log_2_*x*_*G*_, log_2_*x*_*R*_) grid in the left graph of Figure 3, we find that the curve-fit normalization warps it and removes the otherwise orthogonal relationship between log_2_*x*_*R *_and log_2_*x*_*G *_(if the (2*A, M*) plane is considered).

### Perpendicular translation normalization revisited

The perpendicular (shift-log) normalization method proposed by [13] corrects for differences in the channel offsets. It normalizes log-ratios using a translation transform where a constant, *a *∈ ℝ, is added to the signals in one channel and subtracted from the other;

$\begin{array}{c} y_{R,i}\leftarrow a_{R}+b_{R}x_{R,i}+a;\;\forall i\\ y_{G,i}\leftarrow a_{G}+b_{G}x_{G,i}-a;\;\forall i. \end{array}\;\left(12\right)$

We refer to this translation normalization transform as the *perpendicular translation normalization*, because it moves (*x*_*G*_, *x*_*R*_) perpendicular to the *x*_*R *_= *x*_*G *_line. From (9), we get that the observed log-ratios *m*_*r*_(*A*) can be made independent of the intensities if and only if

$a=\frac{rb_{R}a_{G}-b_{G}a_{R}}{b_{G}+rb_{R}},\;r>0.\;\left(13\right)$

As this is a function of *r*, it is only for a single fold change at a time this method can make *M *independent of *A*. The most common choice is *r *= 1 for which the optimal perpendicular shift is

**Figure 4:**
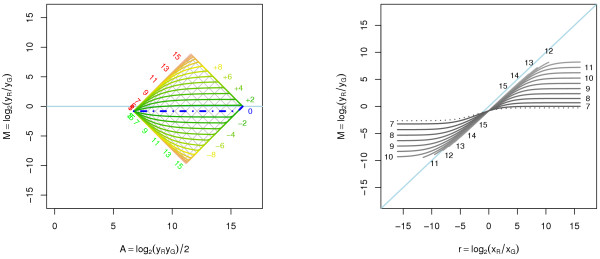
**Perpendicular translation normalization of affine transformed data**. Perpendicular translation normalization of $A_{1}$ transformed data. The optimal amount of normalization shift in the raw data is *a *= 60, which corresponds to ${a^{\prime }}_{R}$ = 80 and ${a^{\prime }}_{G}$ = 140. *Left*: Log-ratios as a function of log-intensities for certain fold changes. The *r *= 1 curve (dot-dash blue) is horizontal, that is, for this specific value of *r *and *a *the log-ratios are independent of the log-intensities. *Right*: Normalized log-ratios versus true log-ratios. From this graph it is clear that we obtain the minimum error in log-ratios at zero-fold change. The dotted curves correspond to the minimum and maximum log-intensities possible to observe.

$a=\frac{b_{R}a_{G}-b_{G}a_{R}}{b_{G}+b_{R}},\;\left(14\right)$

which is the weighted difference between *a*_*R *_and *a*_*G *_with weights *b*_*G*_/(*b*_*G *_+ *b*_*R*_) and *b*_*R*_/(*b*_*G *_+ *b*_*R*_), respectively. The distance from the *r *= 1 curve to the *M *= 0 curve for the optimal perpendicular shift is log_2 _*β*. In other words, the perpendicular shift normalization will not remove an overall bias in the log-ratios (although it is not hard to estimate *β *afterward). The optimal shift for $A_{1}$ is *a *= 60 with log_2_*β *= 0.57. The result of this normalization is depicted in Figure 4. Note that *m*_*r*_(*A*) after normalization is constant for *r *= 1.

As suggested by [13], one way to find the optimal shift *a *is to minimize the curvature by minimizing the variation of the log-ratios after applying the shift *a*. To do this robustly, the median absolute deviation (MAD) can be used as a measure of variation;

$\hat{a}=\arg\underset{a}{\min}\underset{1\leq i\leq I}{\text{MAD}}(M_{i}(a)).\;\left(15\right)$

We have found that the variance of $\hat{a}$ is unnecessarily large.

A problem with the perpendicular translation normalization methods, which is not related to estimator (15), is that the optimal shift can result in non-positive signals making a huge number of expression ratios invalid. The normalization method discussed next does not have this problem, but on the other hand, it will not work or work badly under certain conditions.

### Parallel translation normalization revisited

For historical reasons, but also because it contributes to our discussion about background correction, the shift-log method proposed by [26] for stabilizing (read decreasing or shrinking) the variance of the measured log-ratios is of interest. A side effect of this method is that it can correct for intensity-dependent curvature. It is based on a translation transform where the same constant, *a *∈ ℝ, is added to the signals in both channels;

$\begin{array}{c} y_{R,i}\leftarrow a_{R}+b_{R}x_{R,i}+a;\;\forall i\\ y_{G,i}\leftarrow a_{G}+b_{G}x_{G,i}+a;\;\forall i. \end{array}\;\left(16\right)$

Because (16) moves data (*x*_*G*_, *x*_*R*_) parallel to the *x*_*R *_= *x*_*G *_line, it is referred to as the *parallel translation normalization*. Again, as this is a function of *r*, *M *can only be made independent of *A *for one unique *r *at the time, cf. (9). For *r *= 1 the optimal parallel shift is

**Figure 5:**
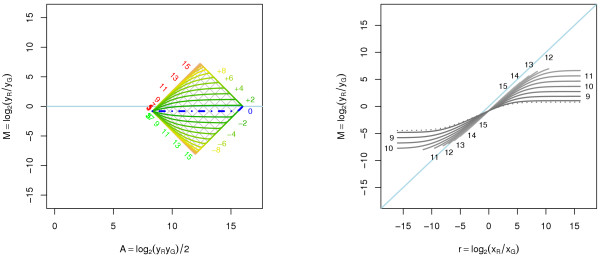
**Parallel translation normalization of affine transformed data**. Parallel translation normalization of $A_{1}$ transformed data. The optimal amount of normalization shift in the raw data is *a *= 220, which corresponds to an effective shift of (${a^{\prime }}_{G}$, ${a^{\prime }}_{R}$) = (420, 240). *Left*: Log-ratios as a function of log-intensities for certain fold changes. The *r *= 1 curve (dot-dash blue) is horizontal, that is, for this specific value of *r *and *a *the log-ratios are independent of the log-intensities. *Right*: Normalized log-ratios versus true log-ratios. From this graph it is clear that we obtain the minimum error in log-ratios at zero-fold change.

$a=\frac{b_{R}a_{G}-b_{G}a_{R}}{b_{G}-b_{R}},\;b_{G}\neq b_{R},\;\left(17\right)$

which may be estimated as in (15). For example, for $A_{1}$ the optimal parallel shift is *a *= 220 with the *r *= 1 curve 0.57 units below the *M *= 0 line. The result of this normalization is depicted in Figure 5. From the above expression, we also see that an optimal value of *a *can indeed be negative. For example, if (*a*_*G*_, *a*_*R*_) = (200,140) and (*b*_*G*_, *b*_*R*_) = (1-4, 0.8), the optimal parallel shift is *a *= -60, which corresponds to an effective shift of (${a^{\prime }}_{G}$, ${a^{\prime }}_{R}$) = (140, 80). However, it can also result in non-positive signals and therefore undefined log-ratios. For example, with (*a*_*G*_, *a*_*R*_) = (20, 200) and (*b*_*G*_, *b*_*R*_) = (1-4, 0.8), the optimal parallel shift is *a *= -440, which corresponds to an effective shift of (${a^{\prime }}_{G}$, ${a^{\prime }}_{R}$) = (-420, -240). Moreover, from (17) we see that when the scale parameters are equal there is no solution. This is because in such cases data is moved in parallel to the *x*_*R *_= *x*_*G *_line making it impossible to get closer. As in the case of the perpendicular shift normalization, the distance between the *r *= 1 curve and the *M *= 0 curve is log_2 _*β*. Hence, a parallel shift normalization will not remove an overall bias in the log-ratios either and rescaling is necessary.

### Single-channel translation normalization

A hybrid of the previous two methods is a normalization method that translates the signals in one of the channels at the time according to

$\begin{array}{l} y_{R,i}\leftarrow a_{R}+b_{R}x_{R,i}+a\cdot I((a\geq 0);\;\forall i\\ y_{G,i}\leftarrow a_{G}+b_{G}x_{G,i}-a\cdot I(a<0);\;\forall i,\;\left(18\right) \end{array}$

where I is the indicator function and *a *∈ ℝ. This will not generate non-positive signals as only positive translations are applied. Moreover, because only one channel is shifted an optimal shift will always be found.

### Rescale normalization

The above translation normalization methods remove curvature for non-differentially by adjusting the offset parameters in *α *= *a*_*R *_- *βa*_*G *_keeping the relative scale *β *fixed. Similarly, if the offset parameters are kept fixed, curvature can be removed by adjusting the relative scale *β*. In [11] we show that the scanner may introduce scale (PMT) insensitive (read *fixed*) biases to the channels. Thus, by adjusting the PMT settings such that the curvature of the pre-scanned data is as small as possible one minimizes |*α*| = |*a*_*R *_- *βa*_*G*_|. Indeed, this strategy may in practice be used by many. However, from above we know that this can equally well be done numerically. It is much more important to adjust the PMT (and laser) settings such that the dynamical range of the signals is as large as possible. Furthermore, as scanner settings are often adjusted for each array separately, there will be a discrepancy between arrays, which in any case has to be normalized for.

### Dye-swap normalization revisited

*Dye-swap normalization*, also known as *reverse labeling *and *paired-slides normalization*, is a balanced experimental design for two-color microarrays that can be used whenever two technically replicated hybridizations are available. Consider an experiment with two sets of cell populations, *A *and *B*, for which relative gene expressions, {*r*_*i*_}_*i*_, are to be investigated. After cDNA is obtained through reverse transcription, the two samples are each split into two identical parts, one which is labeled with a red fluorescent dye and one which is labeled with a green fluorescent dye. The red cDNA cocktail from sample *A *is mixed with the green ditto from sample *B *and co-hybridized to the DNA on the first array. After scanning, expression levels ${\left\{(f_{G_{1}}(x_{B,i}),f_{R_{1}}(x_{A,i}))\right\}}_{i}$ are observed. The same is done for the remaining red-green pair for which ${\left\{(f_{G_{2}}(x_{A,i}),f_{R_{2}}(x_{B,i}))\right\}}_{i}$ are observed. Dropping gene index *i*, the dye-swap normalization suggested by [27] is

$\begin{array}{c} M=\frac{1}{2}(M_{1}+M_{2})\\ =\frac{1}{2}\left(\log_{2}\frac{f_{R_{1}}(x_{A})}{f_{G_{1}}(x_{B})}-\log_{2}\frac{f_{R_{2}}(x_{B})}{f_{G_{2}}(x_{A})}\right)\\ =\frac{1}{2}\left(\log_{2}\frac{f_{R_{1}}(x_{A})}{f_{R_{2}}(x_{B})}+\log_{2}\frac{f_{G_{2}}(x_{A})}{f_{G_{1}}(x_{B})}\right)\\ =\frac{1}{2}({M^{\prime }}_{1}+{M^{\prime }}_{2}) \end{array}\;\left(19\right)$

and similarly for the log-intensities

$\begin{array}{c} A=\frac{1}{2}(A_{1}+A_{2})\\ =\frac{\log_{2}(f_{R_{1}}(x_{A})f_{G_{1}}(x_{B}))+\log_{2}(f_{R_{2}}(x_{B})f_{G_{2}}(x_{A}))}{4}\\ =\frac{\log_{2}(f_{R_{1}}(x_{A})f_{R_{2}}(x_{B}))+\log_{2}(f_{G_{2}}(x_{A})f_{G_{1}}(x_{B}))}{4}\\ =\frac{1}{2}({A^{\prime }}_{1}+{A^{\prime }}_{2}). \end{array}\;\left(20\right)$

Thus, the result of a dye-swap can be written as the average of two "virtual" hybridizations (${A^{\prime }}_{1}$, ${M^{\prime }}_{1}$) and (${A^{\prime }}_{2}$, ${M^{\prime }}_{2}$). Moreover, if (and only if) the measurement functions are equal for each array, that is, $f_{R_{1}}=f_{R_{2}}$ and $f_{G_{1}}=f_{G_{2}}$, then the observed ratios will be identical to the true ratios *for non-differentially expressed genes*. For this to be true for differentially expressed genes we know that they also have to be linear, that is, affine with zero intercept.

Several authors [28,29] have reported that dye-swap normalization does remove curvature, but less successful results have also been reported [30]. To better understand the reasons why and when dye-swap normalization works or not, we dissect the measurement functions *f*_*c *_of the four channels *c *= *R*_1_, *G*_1_, *R*_2_, *G*_2 _into (*v*_*c *_◦ *u*_*c *_◦ *t*_*c *_◦ *s*_*c*_) where *s*_*c *_models the process of all steps up to the step where the (not yet labeled) cDNA sample is obtained, *t*_*c *_models the labeling, *u*_*c *_models the following steps including the hybridization, and *v*_*c *_models the scanning etc. As channel *R*_1 _and *G*_2 _are from sample *A *and the other two are from sample *B*, we know that $s_{R_{1}}=s_{G_{2}}=s_{A}$ and $s_{R_{2}}=s_{G_{1}}=s_{B}$. Furthermore, if the labeling process is well controlled, we can assume that $t_{R_{1}}\approx t_{R_{2}}\approx t_{R}$ and $t_{G_{1}}\approx t_{G_{2}}\approx t_{G}$. When channel *R*_1 _and *G*_1 _are hybridized to array 1 and the other two to array 2 we have that $u_{R_{1}}\approx u_{G_{1}}\approx u_{1}$ and $u_{R_{2}}\approx u_{G_{2}}\approx u_{2}$. Moreover, if the same scanner settings are used for both arrays and everything else is equal, we have that $v_{R_{1}}\approx v_{R_{2}}\approx v_{R}$ and $v_{G_{1}}\approx v_{G_{2}}\approx v_{G}$. The overall measurement functions for the channels are then approximately

$\begin{array}{c} f_{R_{1}}\approx v_{R}\circ u_{1}\circ t_{R}\circ s_{A}\\ f_{G_{1}}\approx v_{G}\circ u_{1}\circ t_{G}\circ s_{B}\\ f_{R_{2}}\approx v_{R}\circ u_{2}\circ t_{R}\circ s_{B}\\ f_{G_{2}}\approx v_{G}\circ u_{2}\circ t_{G}\circ s_{A}. \end{array}\;\left(21\right)$

For the dye-swap normalization to be efficient, we conclude that we must control the process of extracting the RNA etc. to an extent such that we can expect *s*_*A *_≈ *s*_*B*_. Moreover, we must also be able to reproduce hybridizations well enough such that *u*_1 _≈ *u*_2_. If these requirements are met, data will be self-normalized. Turning to the affine model, from (19) we have, if $f_{R_{1}}=f_{R_{2}}$ and $f_{G_{1}}=f_{G_{2}}$. that a dye-swap normalization of affine transformation data gives

$\begin{array}{l} {M^{\prime }}_{1}=\log_{2}\frac{a_{R}+b_{R}x_{A}}{a_{R}+b_{R}x_{B}},\\ {M^{\prime }}_{2}=\log_{2}\frac{a_{G}+b_{G}x_{A}}{a_{G}+b_{G}x_{B}},\;\left(22\right) \end{array}$

and similar for ${A^{\prime }}_{1}$ and ${A^{\prime }}_{2}$. For both virtual arrays, the signals in both channels have undergone identical affine transformations. We know from before that identical transformation in both channels does not introduce curvature for the non-differentially expressed genes and that symmetry between up- and down-regulated genes is preserved, cf. perpendicular and parallel shift normalization. If the offsets in any of the two replicated channels are not equal ($a_{R_{1}}\neq a_{R_{2}}$ or $a_{G_{1}}\neq a_{G_{2}}$), the dye-swap normalization will not work.

The above discussion assumed that the same cell samples have been replicated. If biological replicates are used, an additional source of variability is introduced. However, as long as it is possible to assume that for most genes $x_{A_{1}}\approx x_{A_{2}}$ and $x_{B_{1}}\approx x_{B_{2}}$. dye-swap normalization should still perform well.

In [11] we observed that scanners can introduce channel-specific offsets that are stable over time, i.e. $a_{R_{1}}=a_{R_{2}}$ and $a_{G_{1}}=a_{G_{2}}$. Assume that everything else is perfect, but the PMT is adjusted separately for each array resulting in $b_{R_{1}}/b_{R_{2}}\neq b_{R_{2}}/b_{R_{1}}$ so that (22) is not obtained. This may be a reason why dye-swap normalization sometimes fails.

### Alternative dye-swap normalization

An alternative dye-swap normalization method is to average the observed expression levels *before *taking the logarithm

$\begin{array}{c} M=\log_{2}\frac{(f_{R_{1}}(x_{A})+f_{G_{2}}(x_{A}))/2}{(f_{R_{2}}(x_{B})+f_{G_{1}}(x_{B}))/2}\\ =\log_{2}\frac{f_{R_{1}}(x_{A})+f_{G_{2}}(x_{A})}{f_{R_{2}}(x_{B})+f_{G_{1}}(x_{B})}, \end{array}\;\left(23\right)$

and analogously for *A*. This approach uses the *arithmetic mean *of the observed signals whereas the previous dye-swap method used the *geometric mean*. To be able to say more about the difference between the two approaches, we turn to the affine transformation for which we have

$\begin{array}{c} M=\log_{2}\frac{a^{\prime }+b^{\prime }x_{A}}{a^{\prime }+b^{\prime }x_{B}}\\ A=\frac{\log_{2}(a^{\prime }+b^{\prime }x_{A})(a^{\prime }+b^{\prime }x_{B})}{2} \end{array}\;\left(24\right)$

where *a*' = *a*_*R *_+ *a*_*G *_and *b*' = *b*_*R *_+ *b*_*G*_. Again, we note that the dye-swap method makes the transforms in the resulting two virtual channels equal. Comparing the bias in log-intensities between the geometrical and the arithmetical approaches, for the latter we have

$A_{0}=\log_{2}\frac{a_{R}+a_{G}}{2}\;\left(25\right)$

whereas for the former we have

$A_{0}=\log_{2}\sqrt{a_{R}a_{G}}.\;\left(26\right)$

Because (*a*_*R *_+ *a*_*G*_)/2 ≥ $\sqrt{a_{R}a_{G}}$, we conclude that the log-ratio biases are always larger for arithmetic than geometric dye swap. However, there are other differences too. For instance, if each microarray glass array (the *u*_*c *_functions above) introduces the same offset to both channels and this offset is different between arrays, but otherwise everything else is the same, that is, $a_{R_{2}}=a_{R_{1}}+a$ and $a_{G_{2}}=a_{G_{1}}+a$, then geometric dye-swap fails whereas arithmetic dye-swap succeeds to remove curvature.

### Two-channel quantile normalization

Two-channel or in general multi-channel *quantile normalization *[31,32] is based on and relies on the *assumption that the true gene-expression levels in the two biological samples are approximately equally distributed*. If the measurement functions in the two channels, say *f*_*R *_and *f*_*G*_, are different, then the distributions of the measured signals in the two channels are different even if underlying distributions of true expression levels are identical. By estimating the distributions of the two channels and making them equal, for instance to an average distribution, the log-ratios for the *non-differentially *expressed genes will be unbiased and independent of the intensities. Thus, making the density functions of measured data equal for the two channels is the same as making their transformation functions equal, say to *f*_*RG*_, which makes *M *independent of *A *for non-differentially expressed genes. If *f*_*RG *_could be made linear too, this would be true for all fold changes.

**Figure 6:**
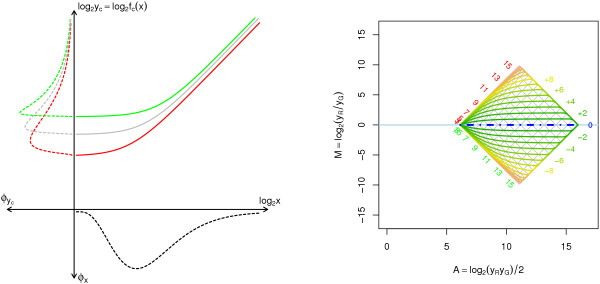
**Equalizing the signal densities of the two channels removes the intensity dependency of the log-ratios for non-differentially expressed genes**. Equalizing the signal densities of the two channels remove the intensity dependency of the log-ratios of non-differentially expressed genes. *Left*: Equal gene-expression distributions in both channels will under the non-channel balanced affine transform $A_{1}$ turn into two different densities for the measured data. The (upside-down and dashed) curve at the bottom shows a hypothetical density function, *φ*_*x*_(·), of the true (log) gene-expression levels expected to be equal in both samples. The distributions of the affine transformed signals are shown in the (rotated and dashed) density functions, ${\left\{\varphi_{y_{c}}(\cdot )\right\}}_{c}$, at the left (red and green curves). The average signal density (middle gray curve) to be normalized toward corresponds to a common measurement function (gray function in the main plot). *Right*: Normalizing the non-equal densities of the two channels makes the log-ratios of the non-differentially expressed genes zero for all intensities.

For affine transformations, two-channel quantile normalization removes intensity-dependent effects, because the offsets *a*_*R*_and *a*_*G *_are identical after normalization. In addition, the constant log-ratio bias log_2_*β *is also removed. Hence, two-channel quantile normalization can be considered to be both a method that corrects for differences in offset between two channels, but also a method that corrects for biases in the expression ratios. In Figure 6, the quantile normalization of $A_{1}$ transformed data is depicted. The curvature for non-differentially expressed genes is removed.

### Background subtraction as a normalization method

**Figure 7:**
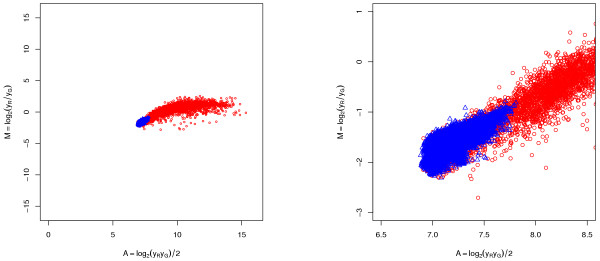
**Transformation of background signal**. Transformation of background signal. *Left*: An *M *versus *A *scatter plot where background signals (blue triangles) and foreground signals (red circles) lye along the same curve, which is evidence that both have been transformed identically. *Right*: A zoom-in of the left graph. Data is from [50].

We have observed that log-ratios of *background signals *show the same intensity-dependent effects as ditto for *foreground signals *do, which suggests that background signals undergo the same transformation as foreground signals. An example of this is shown in Figure 7, where background and foreground estimates are plotted in the same *M *versus *A *scatter plots. A probable reason for this is the existence of scanner biases [11]. A widely adopted rationale for background correction is the assumption that the region that defines the spot is contaminated with the same physical noise that can be observed in the surrounding regions. Background noise is believed to be due to dust particles, DNA contaminated buffers, failed washing during printing or hybridization, cross hybridization etc. [20,33]. This type of background noise is often assumed to add to the foreground signal. Thus, in order to obtain true signals, background is subtracted from foreground signal as

$y_{c,i}\leftarrow y_{c,i}^{\left(\text{fg}\right)}-y_{c,i}^{\left(\text{bg}\right)}\;\left(27\right)$

where $y_{c,i}^{\left(\text{fg}\right)}$ is the estimated foreground signal and $y_{c,i}^{\left(\text{bg}\right)}$ is the estimated background signal for channel *c *and spot *i*. Under a transformation that is dominated by an affine function at lower intensities (of the same level as the background), subtracting the background from the foreground will shift the biases toward zero and background subtracted signals will have less curvature in the (*A*,*M*) plane than non-background subtracted signals (not shown). In this sense we can consider background subtraction to be a normalization method. However, just because the log-ratios as a function of the log-intensities become more flat, it does not imply that foreground regions are contaminated by the same noise as in background regions; unnecessary noise may be introduced. Instead, it may be that the background estimates from the image analysis *happen *to be close to a non-image-related offset in the foreground signals. Moreover, different image analysis software estimate the background signal differently based on different algorithms such as fixed-size circles, adaptive circles, morphological estimates, and pixel intensity distributions. Although comparative studies have been conducted [10,34], it is still not clear which background estimate is most correct. Some methods give higher background estimates than others, which means that they all correct for channel biases by different amounts, which by the way is another argument for why there exist channel offsets. makes use of this is [20], which emphasizes that the true signal can *not *be negative and uses a Bayesian approach to correct for this.

### Result of a (relative) negative translation

**Figure 8:**
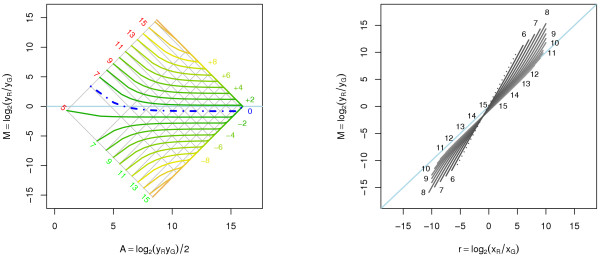
**Affine transformation with negative translation**. Affine transformation of the red and the green signals with negative translation where (*a*_*G*_, *a*_*R*_) = (-87, -24), (*b*_*G*_,*b*_*R*_) = (1.4,0.8). *Left*: Log-ratios as a function of log-intensities for certain fold changes. *Right*: Translated log-ratios versus true log-ratios. The slope of a line fitted in the *M *versus *M *plot will be *larger *than one, which is due to the negative translation. The grid and the fold-change curves in the left graph, and the intensity curves in the right graph have been truncated such that *x*_*R*_,*x*_*G *_≥ 1.

If too much background is subtracted, or a threshold has to be passed before the reverse transcription takes place, one can imagine that *a*_*G*_, *a*_*R *_< 0. Negative bias also applies if the observed signals are compared, not to the true signals, but to the signals obtained by another measuring technique that has a larger bias. Examples of such comparisons can be two-color microarray data compared to oligonucleotide (Affymetrix) data or two-color microarray data compared to QRT-PCR data. Negative bias may also be observed when control clones, spike-ins, negative and positive controls etc. are compared to the genes/ESTs of interest. The effect of a negative translation is depicted in Figure 8. The fan-out effect in the fold-change curves for the lower intensities is due to the negative translation. Note that this should not be mistaken for the fan-out effect due to decreasing signal-to-noise levels in the same way as lack of a fan-out effect due to a positive offset should not be mistaken for low noise.

**Figure 9:**
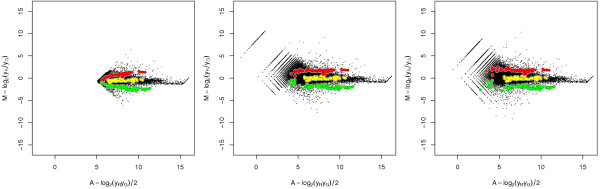
**Log-ratios versus log-intensities before and after a robust affine normalization**. Log-ratios versus log-intensities before and after a robust affine normalization. *Left*: Non-normalized data. Spike-ins designed to have log_2_*r *= +2, 0, and -2 are highlighted in red, yellow and green, respectively. *Middle*: Affine normalization utilizing constraint (32) resulting in no negative signals. Parameter estimates used in back transformation are (${\hat{a}}_{G}$, ${\hat{a}}_{R}$, log_2_$\hat{\beta }$) = (39.0, 22.0, -0.418). *Right*: Affine normalization where 5% (default) negative signals has been allowed; Parameter estimates used in back transformation are (${\hat{a}}_{G}$, ${\hat{a}}_{R}$, log_2_$\hat{\beta }$) = (45.7, 27.0, -0.418). The rotated binning effects of data points at low intensities are due to (unnecessary) rounding of average spot pixel intensity to nearest integer by the image analysis software.

### Robust affine normalization

From the above discussion, it is clear that it is essential to correct for channel offsets when normalizing gene expression data. For two-channel data, we can obtain estimates of *a*_*R*_, *a*_*G *_and *β *as follows. For non-differentially expressed genes (without noise) we have that

*y*_*R,i *_= *α *+ *βy*_*G,i*_; ∀*i *    (28)

with *α *= *a*_*R *_- *βa*_*G *_and *β *= *b*_*R*_/*b*_*G*_. Define $y={\left\{y_{i}\right\}}_{i=1}^{I}$ where **y**_*i *_= (*y*_*G,i*_, *y*_*R,i*_) and let

$Q(\alpha ,\beta ;y)=\sum_{i=1}^{I}w_{i}d_{i}{\left(\alpha ,\beta ;y_{i}\right)}^{2}\;\left(29\right)$

be our objective function where *d*_*i*_(*α*, *β*;**y**_*i*_) > 0 is the orthogonal Euclidean distance between **y**_*i *_and the line *L*(*α*, *β*) with intercept *α *and slope *β*. The estimates of *α *and *β *are then

$(\hat{\alpha },\hat{\beta })=\arg\underset{\left(\alpha ,\beta \right)}{\min}Q(\alpha ,\beta ;y).\;\left(30\right)$

With *w*_*i *_= 1 for all observations we obtain standard principal component analysis (PCA), which minimizes the orthogonal distances in the *L*_2 _norm [35]. With *w*_*i *_≠ 1, (sample-) weighted PCA (WPCA), a special case of generalized PCA, is obtained [35,36]. With weights *w*_*i *_= 1/(*d*_*i*_($\hat{\alpha }$, $\hat{\beta }$; **y**_*i*_) + *δ*) we can minimize the distances in the *L*_1 _norm, if we let *δ *→ 0^+^. The distance *d*_*i *_($\hat{\alpha }$, $\hat{\beta }$; **y**_*i*_), which equals the sum of squares of the values of all but the first principal component, was first suggested by [37]. Thus, our choice of weight function down-weigh outliers as defined by [37] in order to obtain a robust estimate of *L*(*α*, *β*) corresponding to the first principal component. Our procedure is related to principal component analysis applied to an M-estimator of the covariance (scatter) matrix of data. The main difference is that we use weights *w *= *w*(*d*_*i*_) = 1/ (*δ *+ *d*_*i*_) based on the orthogonal distance *d*_*i *_from **y**_*i *_to *L*(*α*, *β*) whereas for M-estimation one uses weights *w *= *w*(*d*_*i*_) based on a robustified Mahalanobis distance of **y**_*i*_, which is computed from an M-estimator of the covariance matrix of data. M-estimation of location and scatter was first defined by [38], and subsequently applied to principal component analysis by [39]. For other more recent papers on robust multivariate analysis, see [40,41] and the references therein. Alternative robust estimators can be obtained by choosing other weight functions *w*(*d*_*i*_), but we choose to optimize in *L*_1_. Moreover, if one suspects a non-symmetric distribution of data points around the line, a trimmed version of the weight function may be considered. In practice, the above optimization can be performed by an *iterative reweighted principal component analysis *(IWPCA) scheme. For iteration *l *= 1,2,..., minimize (29) using WPCA where $w_{i}^{\left(1\right)}$ = 1 and $w_{i}^{\left(l+1\right)}$ = 1/(*d*_*i*_(*α*^(*l*)^,*β*^(*l*)^;**y**_*i*_) + *δ*) with *δ *being a small positive number to avoid infinite weights.

As a last step, in order to get estimates of the four parameters *a*_*R*_, *a*_*G*_, *b*_*R*_, and *b*_*G *_from the two parameter estimates $\hat{\alpha }$ and $\hat{\beta }$, we introduce additional constraints. Let *y*_*c*,(1) _= min_*i*_*y*_*c,i *_for *c *= {*R, G*} and choose

$\begin{array}{l} {\hat{b}}_{G}=1\\ {\hat{b}}_{R}=\hat{\beta }\;\left(\text{31}\right) \end{array}$

$\begin{array}{c} {\hat{a}}_{G}=\max\{a_{G};a_{G}<y_{G,(1)}\wedge \hat{\alpha }+\hat{\beta }a_{G}<y_{R,(1)}\}\\ {\hat{\alpha }}_{R}=\hat{\alpha }+\hat{\beta }{\hat{a}}_{G} \end{array}\;\left(32\right)$

to be the estimates of the bias and the scale parameters in model (3). Constraint (32) is only correct in the noise-free case. If we allow noise, say

*y*_*c,i *_= *a*_*c *_+ *b*_*c*_*x*_*c,i *_+ *ε*_*c,i*_,     (33)

where *E*[*ε*_*c,i*_] = 0 and *V*[*ε*_*c,i*_] = $\sigma_{c,i}^{2}$ for *c *= {*R, G*}, it is possible that the bias terms *a*_*R *_and *a*_*G *_are larger than the smallest observed value in the respective channel. This is especially important if the distributions of *ε*_*c,i *_for *c *= {*R, G*} have heavy negative tails. An alternative, which introduces negative estimates, is to replace *y*_*c*,(1) _in (32) with *y*_*c*, (*j*) _for some order index (*j*) such that *j *- 1 non-positive signals are obtained in channel *c*. Choosing an optimal value on *j *is currently investigated by the authors, but beyond this article. Furthermore, it has been observed that the noise in each channel is roughly proportional to the signal strength, that is, *σ*_*c,i *_∝ *x*_*c,i*_. Thus, a positive side effect of the above estimation algorithm is that, contrary to have equal weights for all spots (*w*_*i *_= 1), more weight will be given to low-intensity spots compared to high-intensity ones. This makes the method more robust to saturation and other non-linear effects that might occur at high intensities, effects for which classical line fits, which rely on homoscedasticity, would fail. Finally, with backward transformation (4) based on estimates (${\hat{a}}_{G}$, ${\hat{a}}_{R}$, ${\hat{b}}_{G}$, ${\hat{b}}_{R}$), data is translated and rotated such that it falls around the diagonal line that goes through (0, 0) and (1, 1).

To illustrate the affine normalization method we have applied it to six two-color microarray data sets each containing 240 spike-in controls designed to have log_2 _*r *= (-2, 0, +2) at various intensities. See also Methods. These controls were not used to estimate the normalization parameters. As shown in Figure 9, which is for one of the arrays, there is a small curvature for non-differentially expressed genes (and spike-ins) before normalization, a curvature that corresponds to -$\hat{\alpha }$ ≈ +7 > 0 (small positive derivative) at log_2 _*r *= 0, cf. (10). More importantly, the intensity dependent effect is profound for the log_2 _*r *= ± 2 controls. Affine normalization allowing no negative signals removes curvature (*α *≈ 0) for log_2 _*r *= 0, but not for the log_2 _*r *= ± 2 controls, which indicates equal affine transformation in both channels, cf. right graph of Figure 6. If 5% negative signals is allowed, the log-ratios of all controls become roughly independent of intensity, which indicates that the observed signals are proportional to the concentrations of the spike-ins. All six arrays in this study show very similar properties.

#### Generalization to multiple channels and multiple arrays

A multi-dimensional version of the above algorithm can be summarized as follows. Say there are *N *arrays each hybridized with *K *samples (colors) such that there is in total *C *= *NK *channels. Let **y**_*i *_= (*y*_1,*i*_,..., *y*_*K*,*i*_,..., *y*_(*N*-1)*K *+ 1,*i*_,..., *y*_*NK*,*i*_) be the *NK *observations for gene *i*. Thus, {**y**_*i*_}_*i *_spans an *NK*-dimensional space. Analogously to the above two-dimensional procedure, we can fit a robust line *L *through data in ℝ^*NK *^and constrain the estimate of **a **= (a_1_,..., *a*_*NK*_) by enforcing that *a *<**y**_*i*_; ∀*i*, where < is the component-wise inequality. Backward transformation (4) translates and rotates data such that it lies along the diagonal line. By normalizing all arrays at once, signals from all hybridizations are brought onto the same scale and no further, so called, between-slide scale normalization is needed.

To apply the multi-dimensional normalization, the assumption that most genes are non-differentially expressed for *all *possible hybridization/channel pairs must be added. For most experimental setups this is not a problem. For instance, in two-channel microarrays experiments it is common to hybridize one test sample and one reference sample, which is selected such that it does not differ too much from the test sample, to the same array. The same reference is then used between arrays (in either channel). Thus, since each test-reference pair is "close" to each other, all test-test pairs should be approximately "close" to each other too. Alternatively, all reference channels can be normalized together. Then, keeping the reference signals fixed, each test channel is normalized toward the corresponding reference channel.

An implementation of the above algorithm is made available in the R [42] package named *aroma *[43], which is platform independent. In addition, the methods are available as an R plugin [44] for BASE [45]. A typically call is normalizeAffine(rg), which will normalize all arrays and all channels in the microarray object rg at once. The first parameter that has to be specified in the above algorithm is *δ*. However, its value is not critical and we have found that for instance *δ *= 0.02 works well in general and is therefore the default value. The second parameter to be specified is the number of negative signals allowed after normalization. By default the method allows 5% negative signals, but any fraction (or absolute number) of negative signals can be specified. Moreover, the method can be applied to any subsets of genes separately such as print-tip groups, clone groups and spike-ins. Finally, support for datapoint weights has been implemented so that the influence each spot has in the estimation procedure can be specified (not to be mistaken for the iterative weights above). Such weights may for instance be calculated from spot quality measures obtained by image analysis methods.

## Discussion

If we compare the robust affine normalization method with the perpendicular and the parallel translation normalization methods optimized by minimizing the curvature, we find that there are similarities, because minimizing the curvature is identical to finding estimates of the bias parameters along the line *L*(*α*, *β*; **y**). Assuming a pure affine transformation, there are also similarities to the curve-fit method, which fits approximately the same line (curve) through data. The difference is how data is transformed to meet the assumptions. The affine method translates and rescales data in the original domain whereas the curve-fit method operates in a rotated and log-transformed domain.

Moreover, the translation and the curve-fit methods rely on two-dimensional data (log-ratios) and it is not clear how to generalize them to multi-dimensional data, although re-iterative versions such as the cyclic loess [31] and the (multi-dimensional) contrast based method [46] have been suggested. Our affine normalization method is not limited to two-dimensional data, but can be applied to any number of channels, which means that three and four-color microarray data can be normalized as easily as two-color data.

It is interesting to note the close relationship between the quantile and the affine normalization method. In quantile normalization data points are shifted such that the sample densities of both channels are made identical. This results in new measurement functions, which may not be linear (or affine), but for which log-ratios for non-differentially expressed genes are zero. The affine normalization method can be though of as a quantile normalization method with special constraints on the underlying densities. An interesting continuation of the affine method and quantile normalization method is to relax the affine constraint by using other parametric or semi-parametric models. One possibility is to add smoothness constraints to the transformation functions using smoothing splines [25].

In previous sections, we did not discuss the variance stabilizing methods suggested by [12,47,48], which are based on error models that also contain channel-specific bias terms. Thus, those methods do indeed correct for intensity-dependent effects. Because they are based on specific error models and target hypothesis testing of non-differentially expressed genes, but also because they stabilize the log-ratio variances, they do not fit well into the above deterministic discussion. In addition, stabilizing the variance introduces bias for *differentially *expressed genes, which is not useful if absolute expression levels are of interest. However, we do believe that the directions drawn up by their underlying error models are promising.

Moreover, in the spirit of [20], it would be interesting to incorporate an empirical Bayes component to allow for non-positive signals more naturally.

An interesting study on microarray scanner calibration curves was published while submitting this article [19]. From their results on under-estimated log-ratios and propeller-shaped log-ratio versus log-ratio scatter plots, we suspect that they observe nothing but affine transformed signals. It would be of great interest to redo their analysis with affine normalization.

Finally, offset and scale parameters in (3) can be extended to incorporate, say, spatial structures by replacing them with *a*_*c*_(**u**_*i*_) and b_*c*_(**u**_*i*_) where **u**_*i *_= (*u*_*i,x*_, *u*_*i,y*_) is the spatial position of spot *i*.

## Conclusion

We have proposed a robust non-parametric normalization method for affine transformed gene-expression data, which centers and symmetrizes log-ratios at all intensities. Symmetric log-ratios are fundamental for statistical tests on non-differentially expressed genes, typically utilizing t-tests or similar. In addition and contrary to other normalization methods (except quantile normalization), which are exclusively for paired channels, the method applies equally well to multi-array and multi-channel data. We believe that normalization based on affine transformations, such as our proposed IWPCA method, is very promising and has the potential of being used for many microarray applications. However, more comparison with other normalization methods is needed to fully understand its advantages and disadvantages.

## Methods

### Log-ratios as a function of log-intensities

Let *x*_*g *_= *b*_*G*_*x*_*G *_≥ 0. Equation (6) for affine transformations (3) can then be written as

$A=\frac{1}{2}\log_{2}[(a_{R}+r\beta x_{g})(a_{G}+x_{g})]$

with *β *= *b*_*R*_/*b*_*G *_and *r *= *x*_*R*_/*x*_*G*_. After a few steps, one gets that

$x_{g}={\left(r\beta \right)}^{-1}\left(-\frac{1}{2}(a_{R}+r\beta a_{G})+\sqrt{\frac{1}{4}{\left(a_{R}-r\beta a_{G}\right)}^{2}+r\beta 2^{2A}}\right).$

It follows that

$\begin{array}{c} a_{G}+b_{G}x_{G}=a_{G}+x_{g}\\ ={\left(r\beta \right)}^{-1}\left(-\frac{1}{2}\alpha (r)+\sqrt{\frac{1}{4}{\left[\alpha (r)\right]}^{2}+r\beta 2^{2A}}\right)\\ a_{R}+b_{R}x_{R}=a_{R}+r\beta x_{g}\\ =\frac{1}{2}\alpha (r)+\sqrt{\frac{1}{4}{\left[\alpha (r)\right]}^{2}+r\beta 2^{2A}} \end{array}$

with *α*(*r*) = *a*_*R *_- *rβa*_*G*_. Equation (9) follows immediately.

### Data

#### Arrays and hybridization

Six arrays were used in this study. The arrays contain Operon's Human Array-Ready Oligo Sets™ and 240 Stratagene SpotReport™ (Alien and Alien Oligo) control spots with layout of 12-by-4 print-tip groups each containing 25-by-25 spots. In total there are 30000 spots on each array. The arrays were produced by the SWEGENE DNA Microarray Resource Centre, Department of Oncology at Lund University using a MicroGrid II 600R arrayer fitted with MicroSpot 10 K pins (BioRobotics). Arrays were spotted on UltraGAPS™ coated slides (Corning Incorporated). Printing was performed in a temperature (18–20°C) and humidity (44–49% RH) controlled area. After printing was completed, arrays were left in a desiccator to dry for 48 hours, rehydrated for 1 second over steaming water, snap dried on a hot plate (98°C), UV-cross-linked (800 mJ/cm^2^) and subsequently hybridized with various test and reference RNA samples. Samples and Stratagene RNA spikes were labeled, purified and hybridized using Pronto!™ Plus System 6 (Corning Incorporated) according to manufacturer's instructions.

#### Scanning and Image analysis

The arrays were scanned on an Agilent G2505A DNA microarray scanner (Agilent Technologies) at laser power and PMT gain both at 100% and scan resolution 10 *μ*m/pixel. The so called *dark offset *intentionally added to all signals by the Agilent scanner [[49], p. 18] has been uninstalled. Multiscan calibration [11] was not used for this study.

The scanned images (65536 gray scales) were analyzed using the Axon GenePix Pro v4.1.1.40 software (Axon Instruments). The median spot pixel intensity was used for the foreground signal. Background estimates were not considered in this analysis. No spot signals were discarded.

## Authors' contributions

HB drafted the first version of the manuscript. Both authors contributed equally to the study and the final version of the manuscript.
